# Supplementary material for: Causal Relationship between Aging and Anorexia Nervosa: A White-Matter-Microstructure-Mediated Mendelian Randomization Analysis
Source: Biomedicines. 2024 Aug 16;12(8):1874. doi: 10.3390/biomedicines12081874 (PMC11351342; doi:10.3390/biomedicines12081874)
Supplement: Supplementary file 1 [file biomedicines-12-01874-s001.zip › Supplementary Figure/Figure S9/Leaveoneout plot of MR analysis for ubm-b-2092 to AN.pdf]

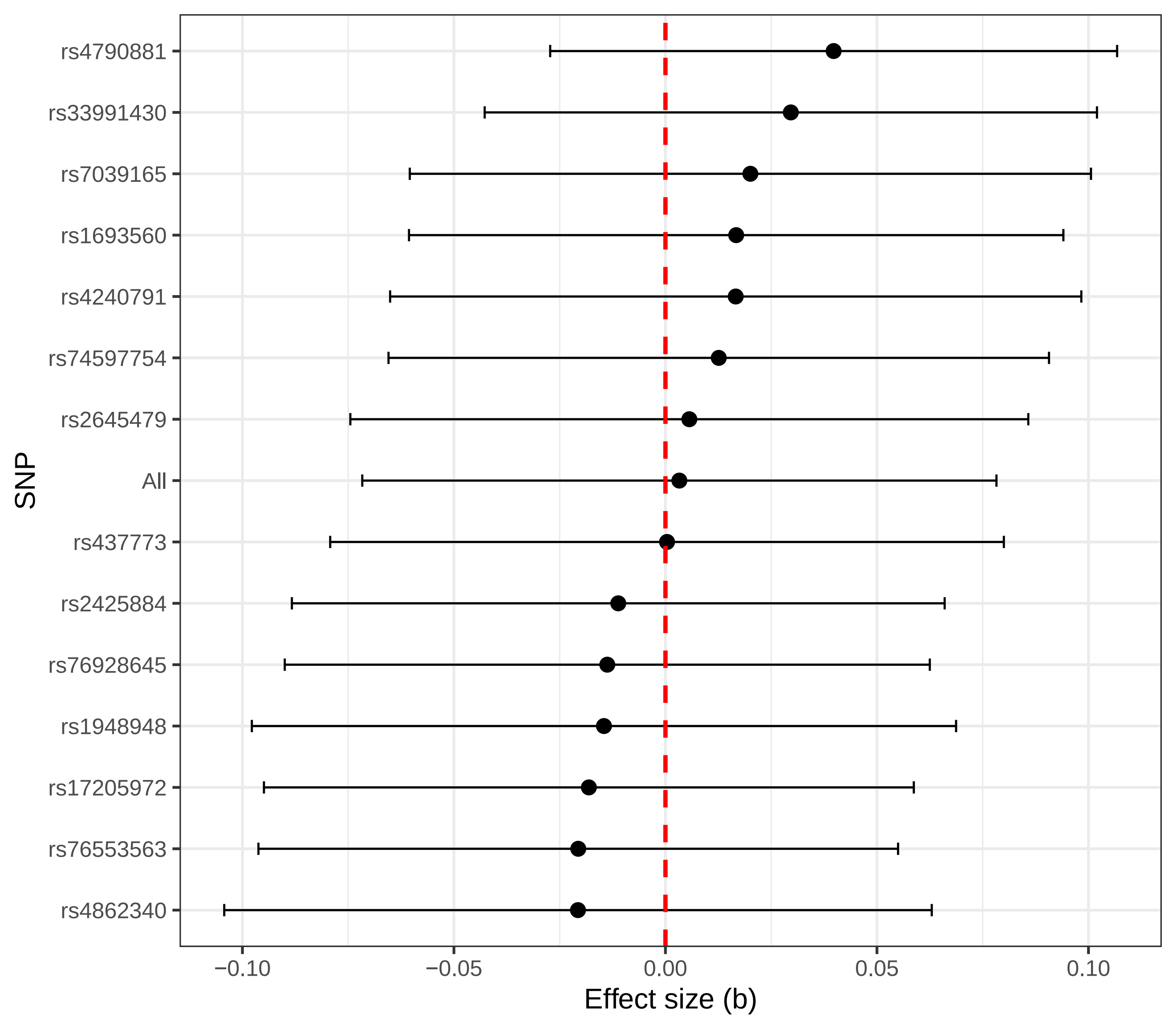

rs4790881
rs33991430
rs7039165
rs1693560
rs4240791
rs74597754
rs2645479
All
rs437773
rs2425884
rs76928645
rs1948948
rs17205972
rs76553563
rs4862340
SNP
−0.10
−0.05
0.00
0.05
0.10
Effect size (b)
